# Suppression of pancreatic cancer liver metastasis by secretion-deficient ITIH5

**DOI:** 10.1038/s41416-020-01093-z

**Published:** 2020-10-07

**Authors:** Eric D. Young, Sharon J. Manley, Thomas C. Beadnell, Alexander E. Shearin, Ken Sasaki, Rosalyn Zimmerman, Evan Kauffman, Carolyn J. Vivian, Aishwarya Parasuram, Tomoo Iwakuma, Paul M. Grandgenett, Michael A. Hollingsworth, Maura O’Neil, Danny R. Welch

**Affiliations:** 1 Department of Cancer Biology, University of Kansas Medical Center, Kansas City, KS USA; 2 Department of Digestive Surgery, Breast and Thyroid Surgery, Kagoshima University, Kagoshima, Japan; 3 Eppley Institute for Cancer Research, University of Nebraska Medical Center, Omaha, NE USA; 4 Department of Pathology and Laboratory Medicine, University of Kansas Medical Center, Kansas City, KS USA

**Keywords:** Metastasis, Cancer models, Pancreatic cancer, Tumour-suppressor proteins

## Abstract

**Background:**

Previously, we identified ITIH5 as a suppressor of pancreatic ductal adenocarcinoma (PDAC) metastasis in experimental models. Expression of ITIH5 correlated with decreased cell motility, invasion and metastasis without significant inhibition of primary tumour growth. Here, we tested whether secretion of ITIH5 is required to suppress liver metastasis and sought to understand the role of ITIH5 in human PDAC.

**Methods:**

We expressed mutant ITIH5 with deletion of the N-terminal secretion sequence (ITIH5Δs) in highly metastatic human PDAC cell lines. We used a human tissue microarray (TMA) to compare ITIH5 levels in uninvolved pancreas, primary and metastatic PDAC.

**Results:**

Secretion-deficient ITIH5Δs was sufficient to suppress liver metastasis. Similar to secreted ITIH5, expression of ITIH5Δs was associated with rounded cell morphology, reduced cell motility and reduction of liver metastasis. Expression of ITIH5 is low in both human primary PDAC and matched metastases.

**Conclusions:**

Metastasis suppression by ITIH5 may be mediated by an intracellular mechanism. In human PDAC, loss of ITIH5 may be an early event and ITIH5-low PDAC cells in primary tumours may be selected for liver metastasis. Further defining the ITIH5-mediated pathway in PDAC could establish future therapeutic exploitation of this biology and reduce morbidity and mortality associated with PDAC metastasis.

## Background

Pancreatic ductal adenocarcinoma (PDAC) is a deadly cancer, due in part to its early and aggressive metastasis to distant organs. Unfortunately, over half of all patients diagnosed with PDAC present with disseminated disease at diagnosis, and 5-year survival is approximately 3%.^1^ Due to hepatic portal circulation, the liver is the most common site of PDAC metastasis. In order to better understand the factors regulating how PDAC spreads to the liver, we designed an in vivo siRNA screen for suppressors of PDAC metastasis and identified inter-α-trypsin-inhibitor-5 (ITIH5) as a suppressor of PDAC metastasis; knockdown of ITIH5 was correlated with increased migration, invasion and liver metastasis.^2^ Much remains to be understood about the role of ITIH5 in both normal physiology and tumour biology. The extracellular matrix protein ITIH5 has been shown to be a tumour suppressor in many cancer types^3–10^ where expression is lost likely due to hypermethylation of the promoter.^11,12^ Compellingly, ITIH5 expression in breast cancer is also found to be associated with a rounded cell morphology^13^ and decreased metastatic efficacy.^14^ However, its mechanism of metastasis suppression in PDAC remains unknown.

Inter-α-trypsin inhibitors (IαI) are secreted proteins with protease inhibitory activity and are thought to play roles in stabilisation of the extracellular matrices.^15–17^ As ITIH5 is also secreted, we hypothesised that ITIH5 secretion would be required for suppression of metastatic outgrowth of PDAC cells in the liver. To test this hypothesis, we generated a construct with deletion of the secretion signal at the N terminus of ITIH5. The non-secreted mutant ITIH5 (ITIH5Δs) was expressed in highly metastatic human PDAC cells (with very low endogenous expression of ITIH5) to examine the effects of ITIH5Δs on PDAC morphology, migration and metastasis. Interestingly, ITIH5Δs, which cannot be secreted (i.e. retained intracellularly), had similar effects as secreted ITIH5 on PDAC cell morphology, motility and suppression of liver metastasis. These data open the possibility that there may be a yet-undescribed role for intracellular ITIH5 in suppression of PDAC liver metastasis. Additionally, we demonstrate reduced ITIH5 levels in human primary PDAC and matched metastases, suggesting that loss of ITIH5 is an early event in PDAC cancer progression.

## Methods

### Cell lines and cell culture

Human PDAC cell lines (SUIT2 derivative S2-007, MiaPaCa-2 and PANC-1) were cultured in DMEM/F12 (Gibco, New York, USA, Cat #11330-032) with 5% foetal bovine serum (FBS) (Atlanta Biologicals, Atlanta, GA, USA, Cat #S11195), 1% glutamine (Gibco, #25030-081), 0.25% non-essential amino acids (Gibco, #11140-050) and 1.5 and 1.0 μg/mL Puromycin (Gibco, #A11138-03) in a humidified incubator at 37 °C. All cell lines were verified free of *Mycoplasma spp*. contamination by a PCR-based assay (ATCC, Manassas, VA, USA, #30-1012K). Cell line identification was authenticated using STR profiling.

### Plasmids and viral transduction

The pReceiver-Lv121 vector (GeneCopoeia, Cat #EX-E1333-Lv121, 10152 bp) was used for viral transduction to express full-length ITIH5 (“ITI” ORF 2829 bp). The N-terminal 18AA secretion sequence of ITIH5 was removed from this construct to create the deleted secretion ITIH5 (ITIH5Δs, “Δs”) mutant. Deletions and restriction sites were inserted using classical directional cloning techniques^18^ and verified by restriction digest and sequencing. Expression of full-length ITIH5 and ITIH5Δs was compared to expression of ITIH5 from control cells expressing control vector (“Con”). Lentiviral transduction was used to introduce the construct into PDAC cells. DNA was amplified using XL10-Gold Ultracompetent Cells (Agilent, Santa Clara, CA, USA, #200315). Plasmids were transfected into packaging cells using 2 µg of DNA, 6 µl of Vira Power Packing Plasmid DNA (ThermoFisher, #K4975-00), and 4 µl of jetPRIME Transfection reagent (Polyplus, Berkeley, CA, USA, #114-15) in 900 µl of Polyplus Buffer (Polyplus, #712-60). This solution was added to packaging cell media for transduction during overnight incubation. An additional 1 ml of media was added to each well of the 6-well plate at 24 h and 48 h after transfection, the viral supernatant was collected, precleared using centrifugation (5’ at 1000 rpm) and filtered through 0.45 low protein-binding filters and applied to cells with 4 μg/ml hexadimethrine bromide (Sigma, St. Louis, MO) at 20–30% confluency. Transductions occurred for 3 days, and then cells rested in culture media before selection in puromycin for 4 days.

### Trypan blue cell proliferation assays

To measure cell proliferation in vitro, 1.0 × 10^4^ human PDAC cells were plated in a 6-well cell culture dish (Corning, Cat #07-200-83) and grown in culture conditions listed above. At days 1, 3, 7, 10 and 14, cells were washed with 1× PBS (Gibco, #10010-023) and detached using trypsin-EDTA (Gibco, #25200-056) until cells were free-floating. Then, an equal volume of media with foetal bovine serum (FBS, Atlanta Biologicals, #S11195) was used to create a single-cell suspension. The concentration of cells was determined using a haemocytometer (Reichert, Depew, NY, USA, Cat #Z359629). Both live and dead cells were counted as distinguished by exclusion or passive uptake of trypan blue solution (Sigma, Cat #T8154). Experiments were conducted in triplicate and standard errors were calculated.

### Cell migration (scratch) assays

PDAC cell lines were cultured using the conditions described above. Cells were plated in 6-well plates (Corning, Corning, NY, USA, Cat #07-200-83) to near confluency after 48 h of growth in a humidified incubator at 37 °C (~1–2 million cells). Plates were scratched using a 200-μl filtered tip (Avant, Valley Park, MO, USA, Cat#ARS-200) with the plate lid as a guide. Two vertical and equally spaced scratches were made in each well. Plates were then rinsed twice with phosphate-buffered saline (PBS), and culture media was replaced before phase-contrast imaging using a Nikon Eclipse TS100 Inverted Microscope, QImaging QIClick monochrome CCD camera and Metamorph^®^ software. Cells were allowed to migrate for 8 h (S2-007) or 72 h (MIAPaCa-2) before reimaging and assessing for closure. Surface area of the open space between cells was measured using ImageJ^®^ software^19^ and the Wound Healing Tool macro.^20^ For each timepoint, three images per scratch and two scratches per experimental group were recorded for each experiment. All experiments were repeated at least three times.

### Immunoprecipitation from conditioned media

Cells were plated and allowed to attach and grow for 48 h. When cells reached approximately 60% confluency, media was replaced, and cells were allowed to grow for 24–48 h. Media was collected in microcentrifuge tubes and spun down at 12,000 rpm for 3 min at 4 °C to remove debris. The supernatant was transferred to a clean microcentrifuge tube (1 ml/sample), and samples were precleared by adding 10 μl protein A/G beads (Santa Cruz, Cat# SC-2003) for 1 h at 4 °C. Samples were centrifuged at 12,000 rpm for 1 min at 4 °C before transferring the supernatant to a new pre-chilled tube. Then, 1 μl of antibody was added to each sample and rotated for 1 h at 4 °C. For control samples, rabbit/mouse IgG (Cell Signaling, Rabbit #2729 or Mouse #5415) was used as appropriate. In all, 20 μl protein A/G beads were added to the mixture, and the sample was incubated with rotation for 4 h overnight at 4°C. Immunoprecipitated proteins were collected using centrifugation at 12,000 rpm for 1 min at 4 °C. The supernatant was aspirated, and beads were washed in 500 μl of ice-cold lysis buffer three times with centrifugation for 1 min at 12,000 rpm and 4 °C. Samples were eluted from the pellet with 20 μl of loading buffer and heated at 95–100 °C for 5 min. Beads were collected by centrifuging at 12,000 rpm for 1 min. The supernatant was transferred to a gel for electrophoresis.

### Immunoblotting

Cells cultured in vitro were lysed using 1× RIPA buffer (Millipore, Burlington, MA, USA, #20-188) with 1% protease-phosphatase inhibitor (Thermo, #78411) on ice for 15 min before centrifuging (13,000 RPM) for 15 min. Protein concentration was determined using a BCA assay (Thermo, #23227). Whole-cell lysates were denatured using NuPAGE LDS Sample Buffer (Invitrogen, Carlsbad, CA, USA, #NP0007) at 95 °C for at least 5 min before separation using either a 12% or 4–20% gradient SDS-PAGE gel (Bio-Rad, Hercules, CA, USA, #5671093) and transferred to PVDF membranes (Bio-Rad, #170-4156), blocked for at least 1 h using 5% non-fat milk in Tris Buffered Saline with Tween-20 (TBST). Antibodies were incubated overnight at 4 °C (ITIH5, ThermoFisher, #PA5-24445) or for 1 h at room temperature (GAPDH, Cell Signaling, Beverly, MA, USA, #2118). A complete list of antibody catalogue numbers is available in Supplemental Table 1.

### Extracellular vesicle isolation

Extracellular vesicle (EV) purification was performed as previously described^21^ with minor modifications. Briefly, cells were grown to 70% confluence at 48 h after plating in 10-cm dishes and washed with serum-free medium. Cells were incubated in 7 ml/dish of serum-free medium or EV-free medium for 36–48 h before harvesting media. Cells were centrifuged in conical tubes in table-top centrifuge with swing bucket rotor at 300 × *g* for 10 min at 4 °C and then transferred to fresh tubes on ice. The supernatant was centrifuged at 2000 × *g* for 10 min at 4 °C. The supernatant was transferred to autoclaved 500-ml Nalgene bottles and centrifuged at 16,500 × *g* for 30 min at 4 °C. The supernatant was transferred to thin polymer open-top tubes (17 ml, Fisher, #03-126) and spun at 110,000 × *g* (24,300 rpm) in an ultracentrifuge using a swing bucket (SureSpin630 6 × 16) rotor for 1.5 h at 4 °C. The supernatant was removed, and pellets were resuspended in 1 ml of ice-cold PBS before centrifuging at 110,000 × *g* (24,300 rpm) using a swing bucket (SureSpin630 6 × 16) rotor for 1.5 h at 4 °C. The supernatant was removed, and the pellet was resuspended in 20–40 μl of PBS before lysis, protein quantification and western blotting. Particle size was measured using a Nanosight^®^ device (settings: frames per second: 24.99, calibration: 189 nm/pixel, blur: auto, detection threshold: 6 multi, min track length: auto, min expected size: 100 nm, temperature: 22.70 °C and viscosity: 0.94 cP) and detected the following particle size: mean: 155 nm, mode: 158 nm and SD: 52 nm.

### Cell fractionation

Cytoplasmic–nuclear fractions were separated according to the manufacturer’s instructions using the NE-PER™ Nuclear and Cytoplasmic Extraction kit (ThermoFisher, Waltham, MA, USA, #78835). Membrane–cytoplasmic fractions were separated according to the manufacturer’s instructions using the Mem-PER™ Plus Membrane Protein Extraction kit (ThermoFisher, #89842).

### Immunocytochemistry

Cells were grown in standard tissue culture conditions and plated onto 8-well chamber slides (Lab-Tek, Grand Rapids, MI, USA, #177402) using culture media. Cells were fixed using 4% paraformaldehyde (PFA) in PBS, blocked with 1% bovine serum albumin (BSA, Fisher, #BP9703-100) in PBS and primary antibodies were applied at 1:100, while secondary antibodies were applied at 1:800 (Alexa 594, #R37117, ThermoFisher). Nuclei were counterstained with DAPI in mounting medium (Vectashield, Burlingame, CA, USA, #H-1200).

### Nuclear localisation sequence identification

Putative nuclear localisation signals were identified using a well-established and highly utilised sequence prediction algorithm available through NLS Mapper software (http://nls-mapper.iab.keio.ac.jp/cgi-bin/NLS_Mapper_form.cgi).^22,23^ Cut-off scores were set at 4.0 searching the entire amino acid sequence in FASTA format.

### Experimental metastasis assay—intrasplenic injections

To measure growth of disseminated pancreatic cancer in liver, PDAC cells were injected intrasplenically into athymic Foxn1^nu^ (Envigo, Indianapolis, IN, USA) mice and allowed to circulate and seed the liver via the portal circulation for 2 min. Next, a splenectomy was performed to remove intrasplenic tumour burden and allow for outgrowth of liver metastases. These procedures were performed as previously described.^2,24^ For S2-007 and MIAPaCa-2 cells, 5 × 10^5^ or 1 × 10^6^ cells were injected into 100 µl of Hanks Balanced Salt Solution (HBSS, Gibco, #14175-103) and allowed to grow for 4 or 8 weeks, respectively, or until the mouse was moribund and required euthanasia. All animal studies were approved by the Institutional Animal Care and Use Committee at the University of Kansas Medical Center (IACUC #2017-2409).

### Immunohistochemistry

Both lungs, all lobes of the liver and PDAC metastases therein were collected during necropsy and fixed in formalin (Fisher, #SF-98-4) overnight before embedding in paraffin. Slides were cut on a microtome (5–7 μm) and deparaffinised before immunohistochemistry (IHC). Antigen retrieval was performed using citrate buffer, pH 6.0 (Thermo, #AP-9003-500) in a steamer for 40 min before cooling and washing with PBS. Peroxidase blocking was achieved using 3% hydrogen peroxide, 5% normal horse serum (Invitrogen, #26050070) and 1% normal goat serum (Invitrogen, #16210064) for protein blocking, and primary antibodies were diluted 1:100 in protein block solution. Secondary antibodies were diluted 1:500 in protein block, and colorimetric reaction was accomplished using diaminobenzidine (DAB, Vector Laboratories #SK-4105). Counterstaining was accomplished using Gill’s haematoxylin #3 (Fisher, #NC9964763), and slides were dried and coverslipped using Permount (Fisher, #SP-15-100).

### IHC quantification

IHC staining for ITIH5 was assessed semi-quantitatively. Aperio AT2 Scanning Microscope (Leica Biosystems, Wetzlar, Germany) was used to scan IHC-stained slides, and Aperio ImageScope software (Leica Biosystems) was used to assess the staining intensity (0+ lowest intensity, 3+ highest intensity) and location (cytoplasmic or nuclear). Areas of interest were identified by two independent observers and validated by a liver pathologist (MO).

### Statistical analysis

Statistical analysis and graphing were done using R software^25^ and Microsoft Excel. Data were normally distributed with equal variance unless otherwise noted. Experimental group means were compared using one-way ANOVA.

## Results

### Expression, localisation and peptide processing of ITIH5 in human PDAC

**Fig. 1:**
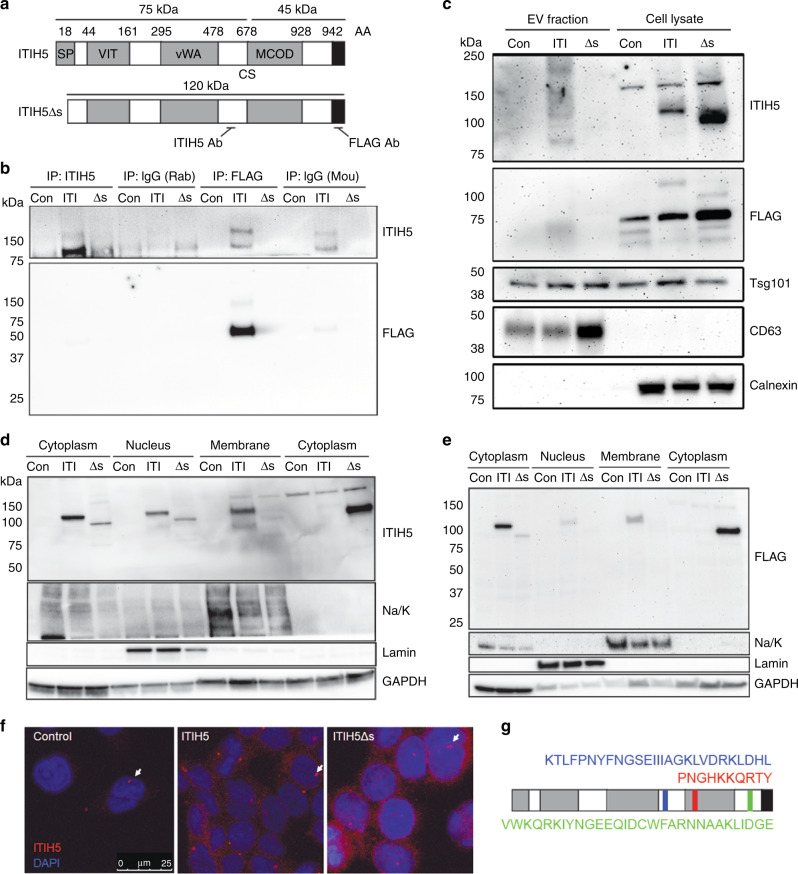
Full-length ITIH5 (ITI) is secreted extracellularly and cleaved in PDAC cells, whereas secretion-deficient ITIH5Δs (Δs) is not secreted extracellularly. **a** Organisation of ITIH5 domain structure labelled by amino acid (AA) position for ITI and Δs expression constructs expressed in the highly metastatic and low endogenous ITIH5-expressing S2-007 human PDAC cell line. Domain map adapted from Himmelfarb et al.^3^ Key features: cleavage site (CS); domains (grey): secretion signal peptide (SP), vault protein inter-α-trypsin domain (VIT), von Willebrand type A domain (vWA), multicopper oxidase domain (MCOD) and FLAG epitope (black). Labelled locations of ITIH5 and FLAG antibody binding. **b** Expression of full-length (ITI) and secretion-deficient ITIH5 (Δs) in S2-007 cells and immunoprecipitation (IP) from conditioned media to detect secreted ITIH5. ITIH5 antibody (binds on N-terminal side of cleavage site at AA578-606) and FLAG antibody (binds on the C-terminal side of CS after the end of ITIH5 open-reading frame). **c** Western blotting of extracellular vesicle (EV) preparations (particle-size range 20–300 nm, mean 155 nm measured using Nanosight^TM^ particle analysis device) for ITIH5. Only minimal ITIH5 is present in EVs. **d**, **e** Western blotting of ITIH5 in cellular fractions. Two separately conducted cellular fractionations are shown per blot where either cytoplasmic fraction was separated from nuclear fraction or membrane/membrane-associated fraction was separated from cytoplasmic fraction. Antibodies used were the N-terminal-detecting ITIH5 (D) or C-terminal-detecting FLAG (E). Extracellularly, large and small fragments of ITIH5 are present. **f** ITIH5 is detected in the cytoplasm and nucleus using immunocytochemistry. Scale bar is 25 µm. **g** Locations of predicted ITIH5 nuclear localisation signals as predicted by sequence recognition algorithm. The algorithm is described in detail.^22,23^ Na/K, sodium/potassium ATPase; GAPDH, glyceraldehyde-3-phosphate dehydrogenase.

IαI family members may be post-translationally cleaved and covalently linked with chondroitin sulfate proteoglycan (CSPG) or very short chains of hyaluronic acid (HA).^15^ Consistent with this pattern, ITIH5 contains a cleavage site (CS) at D681–V686,^3^ predicted to be cleaved by a number of proteases.^15^ The ITIH5 antibody used in our experiments binds an epitope proximal to the CS, while FLAG epitope is at the C terminus of the protein (Fig. 1a). Thus, while both ITIH5 and FLAG antibodies detect uncleaved ITIH5, ITIH5 and FLAG antibodies only detect the C-terminal or N-terminal fragments, respectively (Fig. 1b). Consistent with this reasoning, we detected full-length and uncleaved ITIH5 (~120 kDa) with the ITIH5Δs construct at a slightly lower molecular weight due to the deletion of the 18AA secretion peptide. In the ITIH5 and ITIH5Δs groups, we detected the C-terminal fragment at ~75 kDa (and ~150 kDa when two C-terminal fragments were presumably covalently linked by CSPG^15^), and an N-terminal fragment (~45 kDa) due to 17 AA added after the ORF and a FLAG tag before the STOP codon in the expression construct (Fig. 1b).

We expressed ITIH5 or ITIH5Δs constructs in three highly metastatic human PDAC cell lines that express low endogenous ITIH5: S2-007, MiaPaCa-2 and Panc-1. Control cells express empty vector (“Con”), ITIH5-expressing cells express full-length ITIH5 (“ITI”) and ITIH5Δs cells express secretion-deficient ITIH5 (“Δs”). Levels of ITIH5 overexpression were equivalent to endogenous ITIH5 measured in cultured pancreatic epithelial cells.^2^ As expected, non-secreted ITIH5Δs was not found in conditioned media (Fig. 1b, S2-007 cells shown as representative), suggesting that removal of the secretion peptide prevented ITIH5 from being secreted extracellularly. Next, we looked to see how ITIH5 might be secreted extracellularly by examining the contents of extracellular vesicles (EVs) as ITIH2 and ITIH3 can be secreted in EV.^26^ While both ITIH5 and ITIH5Δs were expressed in the cell lysates (Fig. 1c), very little ITIH5 appeared to be secreted via EV (particle-size range 20–300 nm, mean 155 nm), suggesting that ITIH5 is secreted mainly by an alternative pathway.

To test our hypothesis that ITIH5 secretion would be required for suppression of pancreatic cancer metastasis, we first sought to characterise the intracellular localisation of ITIH5 in PDAC. Using cell fractionation to separate cytoplasmic from nuclear or membrane fractions, we found that ITIH5 runs primarily at its full-length molecular weight of 120 kDa as shown using both the ITIH5 and FLAG antibodies (Fig. 1d, e). However, there are also detectable levels of ITIH5 that run at ~150 kDa, suggesting that ITIH5 is cleaved intracellularly, and that two 75-kDa fragments may be linked by a short chain of CSPG or hyaluronic acid. When cytoplasmic and membrane fractions were separated, we observed consistently less ITIH5Δs in the membrane fraction (Fig. 1d, e). While not formally tested here, this reduction in the amount of ITIH5Δs in the membrane fraction could be due to decreased trafficking through the *trans*-Golgi causing reduced association with the membrane fraction, and/or decreased association with other membrane-associated proteins. Cytoplasmic and nuclear ITIH5 were also observed by confocal immunofluorescence microscopy (IF) (Fig. 1f). Data suggesting nuclear localisation of ITIH5 were supported by the identification of three predicted nuclear localisation signals for ITIH5, as predicted by amino acid sequence at 500–526AA, 731–741AA and 889–918AA (Fig. 1g). Which of these sequences might be responsible for nuclear localisation of ITIH5/ITIH5Δs and the biological significance of nuclear ITIH5/ITIH5Δs is not yet understood and was not assessed in this report.

### Expression of ITIH5 affects cell motility and morphology

**Fig. 2:**
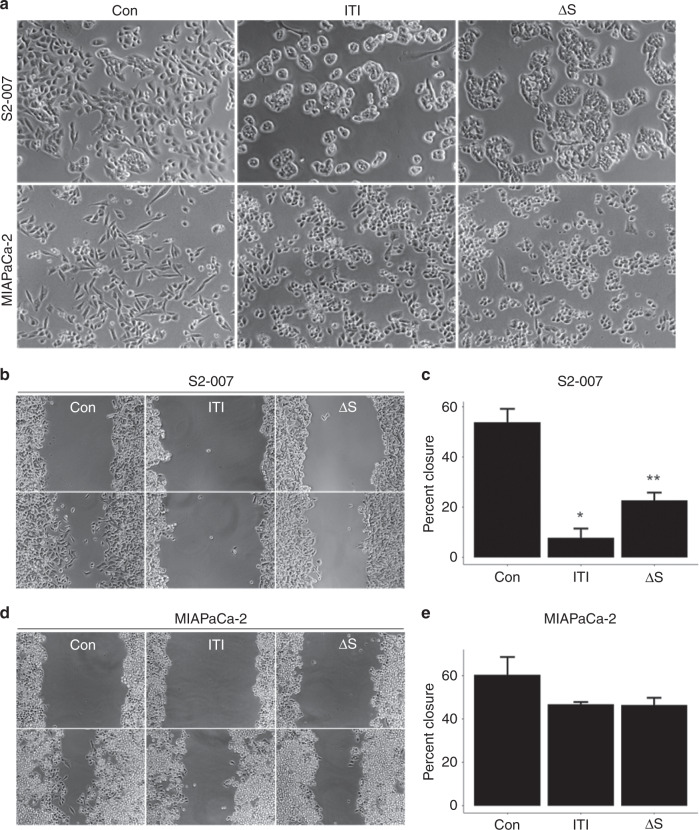
Expression of ITIH5 changes cell morphology and migratory capacity. **a** Expression of full-length ITIH5 and secretion-deficient ITIH5Δs in metastatic human PDAC cell lines (S2-007 and MIAPaCa-2) correlates with rounded cell morphology and tight epithelial-like clustering. Expression of both ITIH5 and ITIH5Δs slowed cell motility in an in vitro wound healing/scratch assay S2-007, 8 h (**b**) and MIAPaCa-2, 72 h (**d**). **c**, **e** Quantification of the results from wound-healing assay measuring the percent of wound closure in S2-007 (**c**) and MIAPaCa-2 (**e**). * and ** S2-007 ITIH5 *P < 0.001 or ITIH5Δs **P < 0.001 compared to control (Con) using Tukey’s test. Error bars show standard error. For MIAPaCa-2 cells, trends were similar although differences were not statistically significant.

Expression of ITIH5 can alter cell morphology in breast cancer.^14^ We observed similar changes in cell morphology in human PDAC after expressing either ITIH5 or non-secreted ITIH5Δs in three metastatic human PDAC cell lines (S2-007, MIAPaCa-2 and Panc-1). All three of these cell lines share activating mutations in KRAS, inactivating mutations in p53 and inactivating mutations in p16 (CDKN2A).^27^ Expression of ITIH5 was associated with a rounded cell morphology and dense aggregation of both S2-007 and MIAPaCa-2 human PDAC cells compared to control cells, with effects in cells with the highest metastatic efficacy (S2-007) being most dramatic (Fig. 2a). Previously, we showed that expression of ITIH5 was correlated with decreased cell motility.^2^ We tested the effect of expressing non-secreted ITIH5Δs on cell motility and found that intracellular ITIH5 also slowed coordinated cell motility in S2-007 (*P* = 1.52e−7, Fig. 2b, c), although the effects in MIAPaCa-2 were not statistically significant (Fig. 2d, e). The results measuring expression with respect to cell morphology and motility in Panc-1 were similar but less pronounced (Supplemental Fig. 1a). We suspect that the reason we were unable to detect changes in cell migration rate in MIAPaCa-2 and Panc-1 is that these cell lines have an already slow rate of cell migration in wound-healing assays. To confirm that this effect was truly a decrease in cell motility—and to reduce the likelihood that observed differences were due to ancillary processes such as decreased rate of proliferation or increased rate of cell death—we measured proliferation in vitro and found that expression of either ITIH5 or ITIH5Δs did not significantly proliferate cell death in any of the three human PDAC cell lines tested (S2-007, MIAPaCa-2 or Panc-1, Supplemental Fig. 1b).

**Fig. 3:**
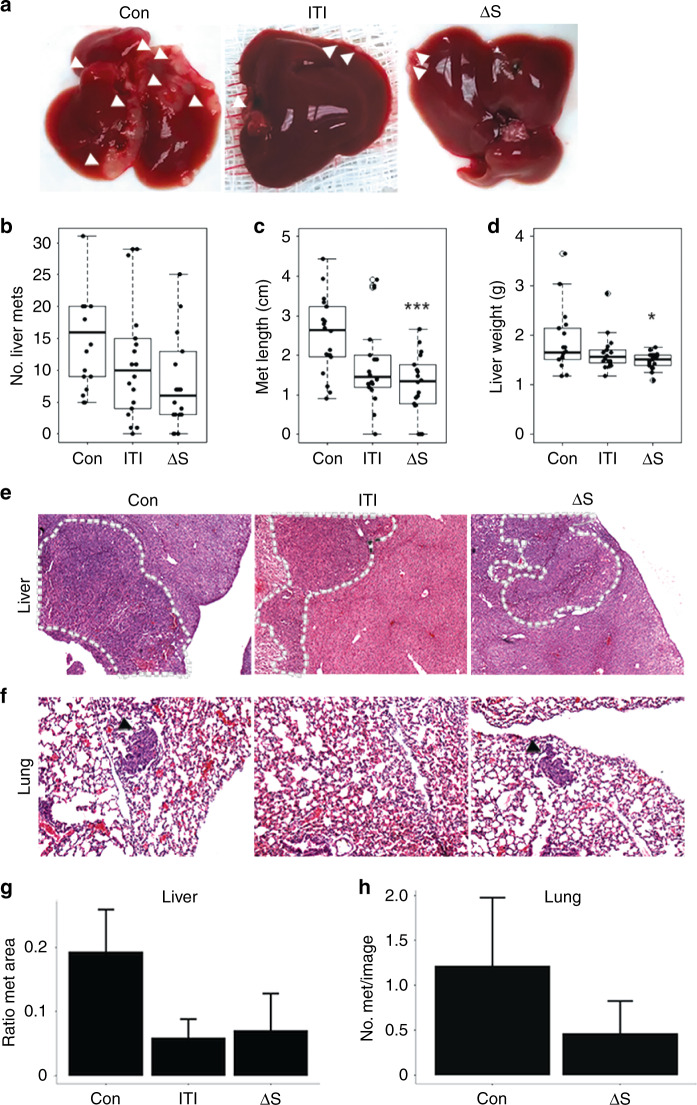
Expression of full-length ITIH5 or secretion-deficient ITIH5Δs suppresses liver metastasis. **a** Representative images of livers following intrasplenic injection of human PDAC cells (S2-007) transfected with control vector (Con), ITIH5 (ITI) or ITIH5Δs (Δs). White arrows highlight liver metastases. **b** Quantification of gross liver metastases from Con, ITI and Δs (median: 16, 10 and 6, respectively). **c** Quantification of metastasis size (mean length (cm) of the longest dimension of gross liver metastases) from Con, ITI and Δs (median: 2.65, 1.45 and 1.35, respectively) ***P < 0.001. **d** Quantification of liver metastasis (by liver weight) from Con, ITI and Δs (median: 1.66, 1.56 and 1.51, respectively). *P = 0.028. **e** Photomicrograph of representative H&E-stained sections of liver with metastases (dashed lines). **f** Photomicrograph of representative H&E-stained sections of lung from mice in (**a**, **e**). Black arrows highlight lung metastases. No lung metastases were identified in the ITIH5-injected mice. **g** Ratio of the area of liver metastases compared to the total histologic liver area. No significant differences. **h** Number of lung metastases in Con and Δs mice (ITI group not shown since there were no lung metastases). No significant differences. Error bars show standard error.

Next, we tested whether secretion of ITIH5 was necessary for suppression of liver metastasis. We injected metastatic human PDAC S2-007 cells expressing ITIH5 or ITIH5Δs into 6- to 8-week-old nude mice intrasplenically and then allowed the cells to seed the liver via the portal vein and grow in the liver for 4–8 weeks (*n* = 18 mice per group). Intriguingly, non-secreted ITIH5Δs also suppressed liver metastasis (Fig. 3a). Animals bearing disseminated tumours expressing ITIH5 or ITIH5Δs showed reduced number and size (measured by the longest dimension) of gross metastases as well as a reduced liver weight (Fig. 3b–d). ITIH5- and ITIH5Δs-expressing cells developed approximately half the number of grossly visible metastases per liver compared to control mice (means: Con = 22.3, ITI = 11.4 and Δs = 9.17. One-way ANOVA, *P* = 0.0455). Similarly, ITIH5 and ITIH5Δs groups showed reduced average size of liver metastases (means: Con = 2.50 mm, ITI = 1.76 mm and Δs = 1.26 mm. One-way ANOVA, *P* = 0.0012). In addition to showing a statistically significant difference in liver metastasis size, the ITIH5Δs group showed a statistically significant reduction in liver weight compared to control (means: Con = 1.877 g, ITI = 1.643 g and Δs = 1.492 g. One-way ANOVA, *P* = 0.0357). These data suggest an intracellular role for ITIH5 in mediating suppression of liver metastasis. We next assessed livers and lungs from these mice (*n* = 3 mice per group) histopathologically. All groups had microscopic liver metastases visible on histopathology (Fig. 3e), with ITIH5 and ITIH5Δs groups showing less liver area occupied by metastases histologically as compared to controls. Those results were not statistically significant (average ratio metastatic area/total liver area, Con = 0.193, ITI = 0.059, Δs = 0.071. One-way ANOVA, *P* > 0.05). Only control and ITIH5Δs groups had detected micrometastases in the lungs (Fig. 3f, average metastases per image, Con = 1.21, Δs = 0.47, Student’s *t* test, *P* > 0.05). No gross metastases were visible on the surface of the lungs. Failure to detect lung metastases in the ITIH5-injected mice does not exclude the possibility that full-length ITIH5 is more effective in suppressing lung metastasis than secretion-deficient ITIH5Δs. However, the most likely interpretation is that the sample size was too small due to the low frequency of lung metastases formed by parental (control) PDAC cell lines. In addition to S2-007 cell lines, in vivo liver metastasis experiments were also performed with MIAPaCa-2 cells, but injection of these cells failed to produce grossly visible liver and lung metastases in all groups (data not shown).

### ITIH5 expression is low in metastases in vivo and in human PDAC

**Fig. 4:**
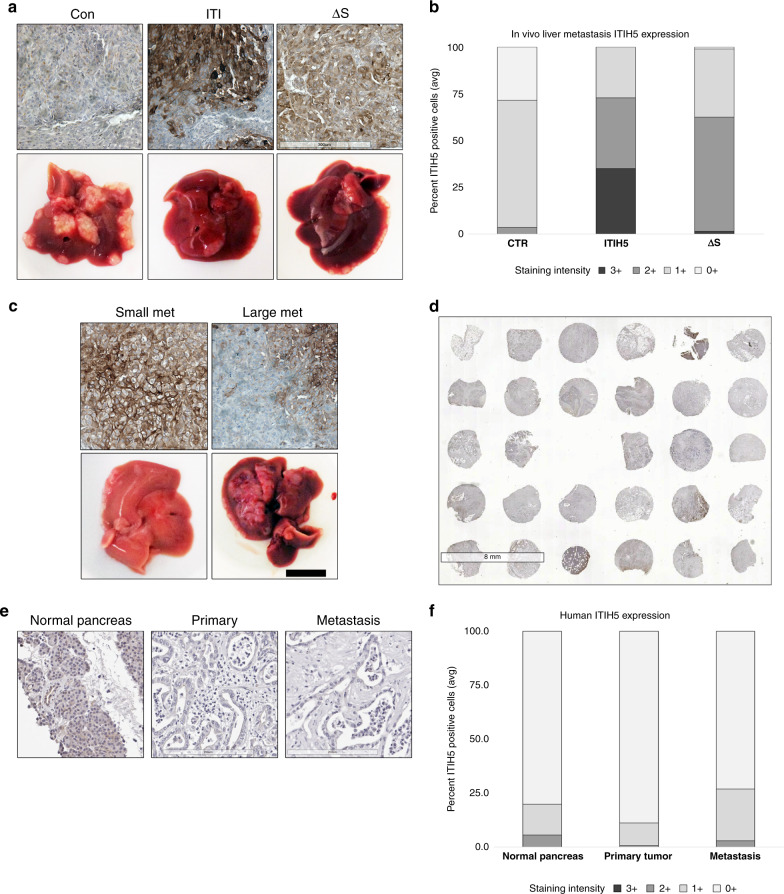
Expression of ITIH5 correlates with reduced liver metastasis in vivo and in human PDAC. **a** Representative images of IHC expression and gross liver images of ITIH5 in control (Con), ITIH5 (ITI) and ITIH5Δs (Δs) expressing PDAC liver metastases. **b** Quantification of photomicrographs of IHC images from in vivo samples shown in (**a**) (*n* = 3). The control group showed frequent low-intensity (0+, 1+) staining, whereas ITI and Δs showed high-intensity (2+, 3+) staining. **c** Representative images of IHC expression for ITIH5 and gross in vivo liver images from tumours expressing full-length ITIH5. Small metastases (small met) expressed higher ITIH5. In contrast, large met had lower (or lost) ITIH5 expression. **d** Representative image of one slide from TMA containing matched human primary and metastatic PDAC and stained for ITIH5 using IHC. ITIH5 expression is generally low in both primary and metastatic PDAC. Focal areas of nonspecific staining of necrotic tissue are also visible. **e** Representative high-magnification images of ITIH5 IHC in normal human pancreas, primary tumour and matched liver metastasis. **f** Quantification of ITIH5 in normal pancreas, primary PDAC and PDAC metastases from TMA samples.

We evaluated protein levels of ITIH5 in liver metastases from mice following intrasplenic injection of S2-007 cells ± expression of ITIH5 or ITIH5Δs by IHC. As anticipated, ITIH5 levels were higher in ITIH5- and ITIH5Δs-expressing tumours than in control tumours (Fig. 4a). ITIH5 or ITIH5Δs staining intensities in liver metastases were high (2+ and 3+) compared to control cells (Fig. 4b). A small number of mice in the ITIH5 and ITIH5Δs groups developed large metastases. When expression of ITIH5 was measured in small and large liver metastases, ITIH5 was high in small metastases (Fig. 4c) but low/lost in large metastases (Fig. 4c).

To further understand clinical relevance, we analysed ONCOMINE data sets and found that expression of ITIH5 mRNA in human PDAC was associated with lower grade, lower stage and with prolonged survival (Supplemental Fig. 1c, d). We used a tissue microarray (TMA) and IHC to assess expression of ITIH5 in human PDAC samples (Fig. 4d). The array contained material from 38 patients, including matched primary and metastasis (*n* = 36 patients), two unmatched human primary and metastatic PDAC samples (*n* = 1 each) and areas of uninvolved human pancreatic duct (*n* = 11). Tissues used as normal controls came from a total of 5 patients. Demographic data of patients included in the TMA are provided in Supplemental Table 2. The demographics of patients who died from PDAC and those who died differ, likely to how their cause of death affected these demographic factors. Patients included as normal controls on the TMA died from the following causes: stroke (2), non-motor vehicle accident (2) and motor vehicle accident (1). Survival duration data were unavailable for patients whose tissues were used as normal controls. In the TMA, IHC for ITIH5 revealed that ITIH5 levels in metastases were similar to those in primary tumours (Fig. 4e). When ITIH5 levels in PDAC tumours were compared to normal pancreatic duct, staining intensity was lower in both primary and metastatic tumours (Fig. 4e), as well as uninvolved normal pancreatic duct (Fig. 4f). These data may suggest that loss of ITIH5 expression may be an early event in PDAC progression, and cells with reduced ITIH5 may be selected for liver metastasis.

## Discussion

ITIH5 is a secreted protein whose loss of expression has been described in many different cancers. In contrast to our original hypothesis that secretion of ITIH5 is required for suppression of liver metastasis, we show that secretion of ITIH5 may not be necessary for suppression of liver metastasis in PDAC. This exciting and unexpected result suggests that there may be a yet-undescribed mechanism for liver metastasis suppression by ITIH5. In contrast to other members of the inter-α-trypsin family,^28–31^ ITIH5 does not appear to be secreted in EVs. While not formally tested here, like other proteins containing N-terminal secretion sequences, ITIH5 may be passed through the endoplasmic reticulum (ER) via co-translational translocation and ultimately through the *trans*-Golgi through the constitutive secretory pathway.^28,32,33^ Removal of the secretion peptide from ITIH5Δs could prevent translocation into the ER and account for the increased cytoplasmic retention observed with ITIH5Δs (Fig. 1d, e).

We showed that expression of both ITIH5 and secretion-deficient ITIH5Δs was correlated with an arrest of cell migration in PDAC. This biology may have a normal physiologic precedent as ITIH5 is highly expressed in the placental syncytiotrophoblast,^2^ and in mice, this expression increases synchronously with arrest of invasion into the endometrium.^34^ Expression of ITIH5 has been associated with transcriptional changes,^13^ and indeed, our data demonstrate that ITIH5 may be present in the nucleus, possibly implicating a role for ITIH5 in such changes.

In the present study, we show a reduction of ITIH5 expression in human PDAC using IHC. We did not, however, observe a further reduction in the levels of ITIH5 in metastatic lesions compared to primary tumours. This could be a limitation of IHC quantification, or it may be that while ITIH5 transcription is reduced in more aggressive tumours, turnover of ITIH5 protein may be less dynamic and would thus fail to show a similar reduction. Here, we provide what we believe to be the first IHC data for expression of ITIH5 in human PDAC. These results suggest that loss of ITIH5 expression is an early event in PDAC progression, and cells with reduced ITIH5 may be selected for liver metastasis. The majority of PDAC cases show liver metastases at diagnosis,^1^ and many patients diagnosed with localised disease eventually develop metastatic disease. Loss of ITIH5 in human PDAC may be one of many critical factors that induce PDAC metastases.

In order to assess experimental liver metastasis, we selected highly aggressive PDAC cell lines even though the intrasplenic model has limitations. For example, while we observed differences in the number and size of liver metastases, we observed no statistically significant differences in the lung metastases between vector control cells and ITIH5-expressing cells. These results could be due to the lung being more hospitable than liver,^35^ rendering growth-suppressive effects of ITIH5 less apparent. Alternatively, the metastasis-suppressing effects of ITIH5 could be liver-specific.

In summary, secretion of ITIH5 is not necessary for liver metastasis suppression in PDAC. There remains much to be understood about the biology of ITIH5 with respect to tumour biology. Understanding this intracellular or cell-autonomous effect of ITIH5 and the pathways involved might help to uncover future therapeutic targets in order to treat patients with disseminated PDAC to the liver and improve the outcome for most patients diagnosed with PDAC. Regardless of our incomplete knowledge of ITIH5 mechanism of action, the data reported here affirm the potential relevance of ITIH5 as a biomarker for assessing tumour progression, metastasis and clinical outcomes for PDAC.

## Supplementary information

Supplemental Materials (Composite)

## Data Availability

Data supporting the results can be found in Supplemental Fig. 1. A comprehensive list of antibodies used in this study can be found in Supplemental Table 1.
