# Supplementary material for: Suppression of pancreatic cancer liver metastasis by secretion-deficient ITIH5
Source: Br J Cancer. 2020 Oct 7;124(1):166–75. doi: 10.1038/s41416-020-01093-z (PMC7782545; doi:10.1038/s41416-020-01093-z)
Supplement: Supplementary file 1 — Supplemental Materials (Composite) [file 41416_2020_1093_MOESM1_ESM.pdf]

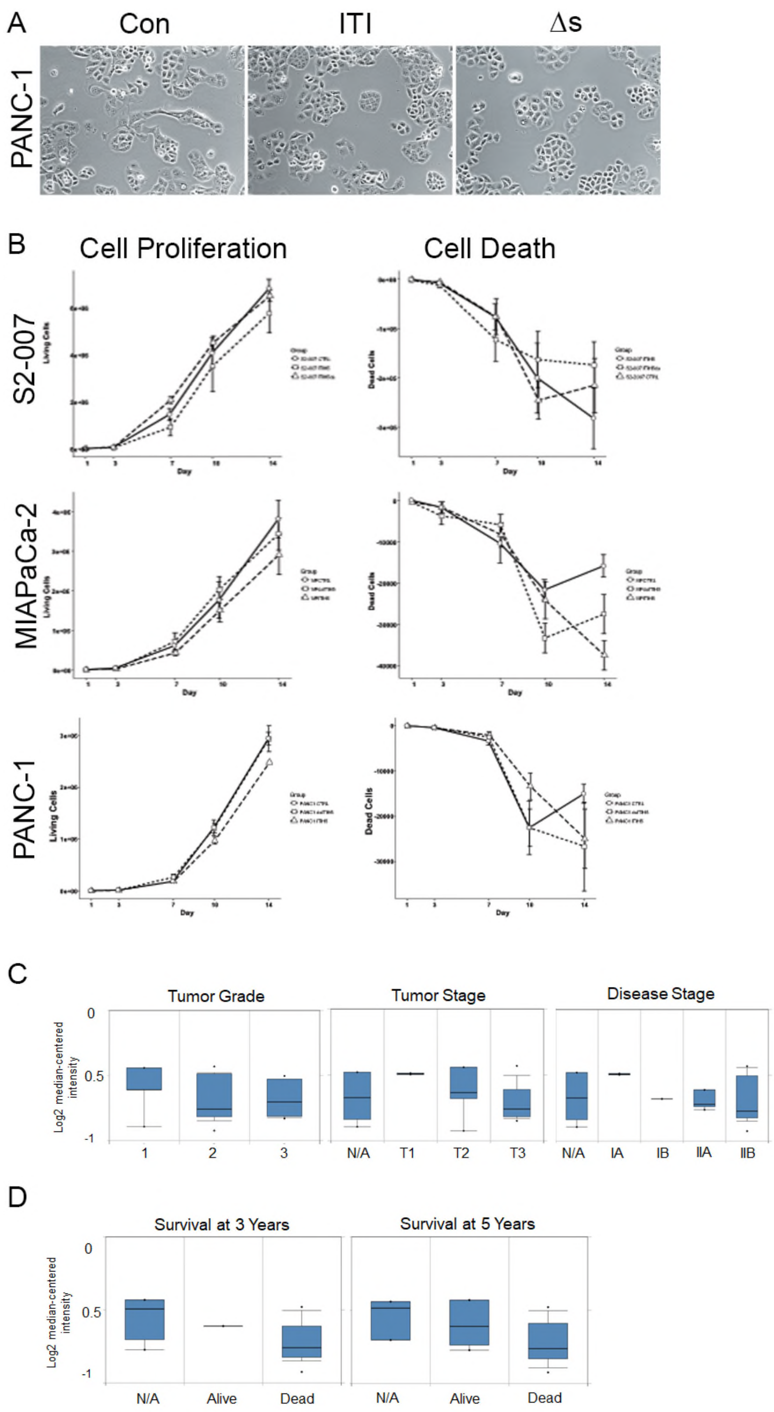

Supplemental Figure 1: A) Representative brightfield photomicrographs of Panc-1 human PDAC cells. Expression of secreted ITIH5 and non-secreted ITIH5Δs alter cell morphology. B) Expression of secreted ITIH5 and non-secreted ITIH5Δs do not change rate of human PDAC cell proliferation or cell death in vitro. C)ITIH5 mRNA expression in human PDAC from ONCOMINE database. High ITIH5 expression correlates with lower tumor grade, stage, and disease stage. D) High ITIH5 mRNA expression from ONCOMINE dataset is associated with increased 3- and 5-year survival in patients with PDAC.

Supplemental Table 1

Names, catalogue numbers, species derivation, antibody type and manufacturer data.

Antibody	Catalogue	Species	Type	Vendor
GAPDH	#2118			Cell Signaling
ITIH5	#PA5-24445	Rabbit	polyclonal	ThermoFisher
NaK Atpase				Santa Cruz
Flag-M2	#F1804-200G	Mouse	monoclonal	ThermoFisher

Supplemental Table 2

Comparison of Demographic Characteristics of Patients Included on Tissue Microarray

	PDAC n=38	Normal Control n= 5
Age, years mean, [range]	66 [49-80]	48.8 [24-65]
Sex, male (percent)	82	60
Height, cm mean, [range]	174 [153-193]	178 [164-194]
Weight, kg mean, [range]	75 [48-130]	93 [69-111]
BMI, mean, [range]	24.9 [16-41]	29.6 [23-37]
Survival, days mean, [range]	333 [9-1,556]	N/A
